# The influence of Apolipoprotein E genotype on regional pathology in Alzheimer’s disease

**DOI:** 10.1186/1471-2377-13-44

**Published:** 2013-05-11

**Authors:** Marwan N Sabbagh, Michael Malek-Ahmadi, Brittany N Dugger, Katarina Lee, Lucia I Sue, Geidy Serrano, Douglas G Walker, Kathryn Davis, Sandra A Jacobson, Thomas G Beach

**Affiliations:** 1 The Cleo Robert Center for Clinical Research, Banner Sun Health Research Institute, Sun City, AZ, USA; 2 Civin Laboratory for Neuropathology, Banner Sun Health Research Institute, Sun City, AZ, USA; 3 Laboratory of Neuroinflammation, Banner Sun Health Research Institute, Sun City, AZ, USA

## Abstract

**Background:**

Carriers of the ApoE ϵ4 allele are at a greater risk for developing Alzheimer’s disease (AD) and those who do develop AD tend to have a much greater neuropathological disease burden. Although several studies have shown significant differences in AD pathology among ϵ4 carriers and non-carriers, few have characterized these differences in terms of brain region and neuropathological score frequency.

**Methods:**

566 pathologically-confirmed AD cases who were followed prospectively with antemortem dementia diagnoses (312 ApoE ϵ4 carriers and 254 ApoE ϵ4 non-carriers) were compared on the frequencies of neuropathological frequency scores (none, sparse, moderate, frequent) among several different brain regions (frontal, temporal, parietal, hippocampal, and entorhinal) using the CERAD scoring system. Pathology score frequencies were analyzed by carrier status (ϵ4 carrier vs. ϵ4 non-carrier) and by genotype (2/3, 3/3, 2/4, 3/4, 4/4). Both analyses investigated pathology score frequencies among different brain regions (frontal, temporal, parietal, hippocampal, and entorhinal).

**Results:**

ϵ4 carriers had a significantly lower age at death (p <0.001) and significantly higher Braak scores (p <0.001) than ϵ4 non-carriers. Genotype comparison revealed that plaque and tangle pathologies increased in the following pattern, 2/3<3/3<2/4<3/4<4/4, for several brain regions. When stratified by age and ApoE ϵ4 carrier status, ϵ4 carriers tended to have significantly more frequent scores across most cortical areas. However, non-carriers age 90 and older tended to have greater plaque pathology than carriers. For tangle pathology, ϵ4 carriers tended to have significantly more “frequent” scores than non-carriers, except for the hippocampal and entorhinal areas in individuals age 90 and older.

**Conclusions:**

ApoE ϵ4 carriers had a significantly higher percentage of “frequent” scores for plaques and tangles when compared to ApoE ϵ4 non-carriers for several brain regions. However, ϵ4 non-carriers age 90 and older tended to have less plaque and tangle pathology in certain brain regions. These results demonstrate that AD pathology may manifest itself differently based on ApoE genotype and suggest that ApoE carriers and non-carriers may have different patterns of AD neuropathology location and density.

## Background

It has been shown that the three main polymorphisms (E2, E3, E4), coded by the ϵ2, ϵ3, and ϵ4 alleles on chromosome 19, of the apolipoprotein E (ApoE) gene result in differential risk factors for Alzheimer’s disease (AD) for those who carry them [1-6]. The ϵ2 isoform, the rarest allele out of the three [7], has demonstrated a protective effect, while subjects heterozygous for the ϵ4 allele experience a three-fold increase in risk for sporadic AD and those homozygous for ϵ4 allele have a 12–18 fold risk increase [5,7,8]. The ϵ4 allele has also been shown to shift the age of onset of AD earlier than those without the allele [8]. Although the ϵ3 allele is the most common allele [9], several studies have shown that the ϵ4 allele is much more frequent among those with late-onset familial and sporadic AD in comparison to controls [10]. The ϵ3 isoform displays an intermediate effect and, due to its higher frequency, its effect on pathologies is taken as the base line comparison for the ϵ2 and ϵ4 isoforms [7].

In addition to being linked to AD, ApoE ϵ4 has also been strongly linked to increased deposition of the main hallmark proteins in AD brains, amyloid beta (Aβ) and neurofibrillary tangles (NFTs), as well as correlating with certain biomarkers found in plasma and cerebrospinal fluid of AD [10,11]. With respect to pathological staging schemes for Aβ deposits and NFTs [12,13] ϵ4 allele carriers tend to be more advanced implying earlier onset of AD [2]. Furthermore, the presence of ApoE ϵ4 allele has been shown to correlate with increased deposition of both amyloid and tau pathology in the neocortex [1]. In non-demented subjects over age 60, ApoE ϵ4 is associated with a higher burden of total parenchymal and vascular amyloid neuropathology than non-demented individuals, but with no difference in tau pathology [3].

The intent of this study is to determine how ApoE genotype affects AD pathology by brain region. By comparing the frequencies of neuropathological scores for several different brain regions, we aim to produce a more refined and specific characterization AD pathology in a large, clinicopathologically confirmed sample that is stratified by age and ApoE genotype.

## Methods

### Subjects

The subjects in this study were recruited from the Banner Sun Health Research Institute Brain and Body Donation Program located in Sun City, Arizona [14]. Individuals enter the program by voluntarily agreeing to brain autopsy after death; persons with dementia are signed into the program by their legal representative. All participants signed informed consent prior to enrolling into the program which was approved by the Banner Health Institutional Review Board (IRB).

Data from 775 cases autopsied between January of 1997 and December 2011 were available for the analysis. Only individuals who had received both a clinical diagnosis of dementia and a neuropathological diagnosis of AD were included in the study. 209 individuals were excluded due to incomplete neuropathological information, other clinical diagnoses of non-AD dementia or unavailable genotype.

The final sample size was 566 and consisted of 312 AD-ApoE4+ and 254 AD-ApoE4- cases who were followed prospectively with antemortem dementia diagnoses. The sample did not differ statistically with respect to numbers of males (n = 295) and females (n = 271). Clinical AD diagnoses were made using DSM-IV criteria [15].

### Pathological assessment

Neuropathological AD diagnoses were made according to National Institute on Aging/Reagan Institute criteria [16] and included those categorized as “intermediate” or “high” probability.

Senile plaque and neurofibrillary tangle load scores were obtained using the CERAD scoring system with separate semi-quantitative density estimates of none, sparse, moderate, or frequent (converted to a 0–3 scale for statistical purposes) using standardized published templates [17]. Regions scored included cortical gray matter from frontal (F), temporal (T), parietal (P), hippocampal CA1 (H), and entorhinal (E) regions. The individual carrying out the scoring (TB) was blinded to demographics and ApoE status. Plaque and tangle scores from the F, T, P, H, and E were compared between ApoE ϵ4 carriers and ApoE ϵ4 non-carriers and also between each individual isoform [12,13].

### ApoE genotyping

DNA for ApoE genotyping from autopsy cases was extracted from pieces of fixed cerebellum tissue. Tissue (100 mg) was digested to completion with proteinase K (1 mg/ml) at 55°C and extracted with phenol/chloroform. DNA was recovered by isopropanol precipitation. For PCR reactions, 500 ng of DNA from each sample was used. PCR primers, amplification conditions employed, and identification of ApoE genotypes by Hha I digestion of amplified material, were carried out according to a published protocol [18]. Digested fragments were separated by electrophoresis through 9% acrylamide gels and identified by staining with fluorescent dye Gel Red (Biotium, Hayward, CA).

### Statistical analysis

For demographic data, chi-square analysis was used to assess the association between ApoE ϵ4 carrier status and gender. The Mann–Whitney U test was used to discern differences on age at death and years of education between ApoE ϵ4 carriers and non-carriers. The effect size of these differences was assessed using Cohen’s *d*.

For the pathological data, the sample was stratified by ApoE ϵ4 carrier status and age with the frequency of pathological score reported for each stratified group. The sample was divided into the following age groups: ≤69, 70–79, 80–89, ≥90. The data were also stratified by ApoE genotype to examine pathological differences between each ApoE isoform. For the stratified carrier status and age analyses, the Mann–Whitney U test was used to compare plaque and tangle rating frequency differences between ApoE ϵ4 carriers and non-carriers in each age group. For the genotype analyses, the Kruskal-Wallis test was used to compare differences of plaque and tangle pathology rating frequencies. Groupwise comparisons were carried out using the Dwass-Steel-Chritchlow-Fligner test.

## Results

For the sample of 566, the average age at death was 82.56 (8.33) years and the average education level was 14.54 (2.67) years. The sample was comprised of 295 males and 271 females. Demographic characteristics broken down by ApoE ϵ4 carrier status are displayed in Table 1. There was no significant association between ApoE ϵ4 carrier status and gender (χ^2^ = 1.17, df (1), p = 0.28). Education was not significantly different between carriers and non-carriers, however ϵ4 carriers had a significantly younger age at death and significantly higher Braak score than non-carriers. The effect size for age at death was small, while the effect size for Braak score was moderate. Table 2 displays the demographic and clinical data for each ApoE genotype.

**Table 1:** Demographic characteristics by ApoE ϵ4 carrier status

	**ApoE ϵ4 carrier**	**ApoE ϵ4 non-carrier**	**p-value**	**Effect size**
n (%)	312 (55)	254 (45)	----	----
Gender (M/F)	169/143	126/128	0.28	----
Age at Death	81.53 (7.49)	83.81 (9.12)	<0.001	0.27
Education	14.52 (2.45)	14.56 (2.91)	0.87	0.01
Braak Stage	5.01 (1.03)	4.53 (1.26)	<0.001	0.42

Mean (sd = standard deviation).

**Table 2:** Demographic characteristics by ApoE genotype

	**ApoE 2/3**	**ApoE 3/3**	**ApoE 2/4**	**ApoE 3/4**	**ApoE 4/4**
n (%)	22 (3.9)	232 (41)	14 (2.5)	238 (42)	60 (10.6)
Gender (M/F)	17/5	109/123	7/7	124/114	38/22
Age at Death	84.64 (9.20)	83.73 (9.13)	84.36 (7.50)	81.63 (7.51)	80.48 (7.34)
Education	15.36 (3.37)	14.49 (2.86)	13.55 (2.73)	14.51 (2.41)	14.91 (2.52)
Braak Stage	4.36 (1.43)	4.55 (1.25)	4.54 (1.39)	4.99 (1.03)	5.19 (0.88)

Mean (sd = standard deviation).

Scores for plaque and tangle frequencies stratified by age group and ApoE ϵ4 carrier status are shown in Tables 3 and 4. In the ≤69 group, no statistically significant differences were found between ApoE ϵ4 carriers and non-carriers for plaques and tangles in all cortical areas. Although the percentages of individuals show that ApoE ϵ4 carriers had more “frequent” scores, the lack of statistical significance is likely due to the small cell counts for this age group. For the 70–79 age group, ApoEϵ4 carriers had significantly more “frequent” plaque density scores than non-carriers in all cortical areas, except for the temporal area. For tangle density scores in the 70–79 age group, the only area to show statistically significant difference was the hippocampal area. In the 80–89 age group, ApoEϵ4 carriers had significantly more “frequent” plaque density scores than non-carriers in all cortical areas for both plaque and tangle densities. For the ≥90 age group, non-carriers had significantly more “frequent” plaque density scores than carriers for all cortical areas except the hippocampal area where carriers had slightly more “frequent” scores than non-carriers. The latter was not statistically different. For tangle densities in the ≥90 age group, ApoEϵ4 carriers had significantly more “frequent” scores for the frontal, temporal, and parietal areas, but significantly fewer “frequent scores” for the hippocampal and entorhinal areas.

**Table 3:** Frequency of plaque pathology scores for ApoE ϵ4 carriers and non-carriers by age group

**Age group**		**≤69**		**70 - 79**		**80 - 89**		**≥90**	
		ϵ4+	ϵ4 -	ϵ4+	ϵ4 -	ϵ4+	ϵ4 -	ϵ4+	ϵ4 -
Frontal Plaque Density	None	0 (0%)	0 (0%)	0 (0%)	0 (0%)	0 (0%)	0 (0%)	0 (0%)	1 (1%)
	Sparse	0 (0%)	0 (0%)	1 (0.7%)	1 (0.7%)	0 (0%)	3 (1.1%)	0 (0%)	1 (1%)
	Moderate	2 (5.1%)	1 (2.6%)	5 (3.6%)	8 (5.8%)	5 (1.8%)	13 (4.6%)	2 (2%)	11 (10.8%)
	Frequent	21 (58.9%)	15 (38.5%)	86 (61.87%)	38 (27.3%)	147 (52.5%)	112 (40%)	38 (37.3%)	49 (48%)
		p = 0.78		p = 0.03		p = 0.003		p = 0.03	
Temporal Plaque Density	None	0 (0%)	0 (0%)	0 (0%)	0 (0%)	0 (0%)	0 (0%)	0 (0%)	0 (0%)
	Sparse	0 (0%)	0 (0%)	0 (0%)	2 (1.4%)	0 (0%)	3 (1.1%)	0 (0%)	2 (2%)
	Moderate	1 (2.6%)	0 (0%)	7 (5.0%)	6 (4.3%)	7 (2.5%)	21 (7.5%)	2 (2%)	8 (7.9%)
	Frequent	22 (56.4%)	16 (41.0%)	85 (61.2%)	39 (28.1%)	146 (52.1%)	103 (36.8%)	38 (37.6%)	51 (50.5%)
		p = 0.40		p = 0.08		p < 0.001		p = 0.08	
Parietal Plaque Density	None	0 (0%)	0 (0%)	0 (0%)	0 (0%)	0 (0%)	0 (0%)	0 (0%)	2 (2%)
	Sparse	0 (0%)	0 (0%)	0 (0%)	1 (0.7%)	0 (0%)	1 (0.4%)	0 (0%)	1 (1%)
	Moderate	0 (0%)	0 (0%)	5 (3.6%)	6 (4.4%)	3 (1.1%)	14 (5%)	1 (1%)	7 (7%)
	Frequent	23 (59%)	16 (41%)	87 (63%)	39 (28.3%)	148 (53.1%)	113 (40.5%)	39 (39%)	50 (50%)
		p = 0.99		p = 0.05		p = 0.001		p = 0.03	
Hippocampal Plaque Density	None	0 (0%)	0 (0%)	2 (1.5%)	7 (5.1%)	3 (1.1%)	11 (3.9%)	0 (0%)	8 (8%)
	Sparse	3 (7.7%)	1 (2.6%)	5 (3.6%)	6 (4.4%)	18 (6.4%)	38 (13.6%)	9 (9%)	15 (15%)
	Moderate	4 (10.3%)	6 (15.4%)	44 (31.9%)	18 (13%)	70 (25%)	41 (14.6%)	15 (15%)	23 (23%)
	Frequent	16 (41%)	9 (23.1%)	40 (29%)	16 (11.6%)	62 (22.1%)	37 (13.2%)	16 (16%)	14 (14%)
		p = 0.56		p = 0.03		p < 0.001		p = 0.02	
Entorhinal Plaque Density	None	0 (0%)	0 (0%)	1 (0.7%)	4 (2.9%)	2 (0.7%)	2 (0.7%)	1 (1%)	3 (3%)
	Sparse	0 (0%)	0 (0%)	1 (0.7%)	5 (3.6%)	3 (1.1%)	13 (4.6%)	1 (1%)	1 (1%)
	Moderate	2 (5.1%)	2 (5.1%)	22 (15.8%)	14 (10.1%)	38 (13.6%)	39 (13.9%)	9 (8.9%)	24 (23.8%)
	Frequent	21 (53.9%)	14 (35.9%)	68 (48.9%)	24 (17.3%)	110 (39.3%)	73 (26.1%)	29 (28.7%)	33 (32.7%)
		p = 0.70		p = 0.002		p = 0.005		p = 0.08	

n (% = percent).

**Table 4:** Frequency of tangle pathology scores for ApoE ϵ4 carriers and non-carriers by age group

**Age group**		**≤69**		**70 - 79**		**80 - 89**		**≥90**	
		ϵ4+	ϵ4 -	ϵ4+	ϵ4 -	ϵ4+	ϵ4 -	ϵ4+	ϵ4 -
Frontal Tangle Density	None	1 (2.6%)	0 (0%)	14 (10.2%)	11 (8%)	20 (7.1%)	36 (12.9%)	6 (5.9%)	19 (18.6%)
	Sparse	1 (2.6%)	0 (0%)	13 (9.4%)	8 (6%)	31 (11.1%)	36 (12.9%)	10 (9.8%)	21 (20.6%)
	Moderate	0 (0%)	0 (0%)	8 (5.8%)	5 (3.6%)	27 (9.6%)	10 (3.6%)	6 (5.9%)	10 (9.8%)
	Frequent	21 (53.9%)	16 (41.3%)	56 (40.9%)	23 (16.7%)	74 (26.4%)	46 (16.4%)	18 (17.7%)	12 (11.8%)
		p = 0.23		p = 0.14		p = 0.001		p = 0.006	
Temporal Tangle Density	None	1 (2.6%)	0 (0%)	8 (5.8%)	8 (5.8%)	9 (3.2%)	13 (4.6%)	1 (1%)	8 (7.9%)
	Sparse	1 (2.6%)	0 (0%)	9 (6.5%)	7 (5.1%)	12 (4.3%)	30 (10.7%)	9 (8.9%)	15 (14.9%)
	Moderate	0 (0%)	0 (0%)	10 (7.3%)	4 (2.9%)	14 (5%)	20 (7.1%)	3 (3%)	12 (11.9%)
	Frequent	21 (53.9%)	16 (41.3%)	64 (46.4%)	28 (20.3%)	118 (42.1%)	64 (22.9%)	27 (26.7%)	26 (25.7%)
		p = 0.46		p = 0.13		p < 0.001		p = 0.02	
Parietal Tangle Density	None	2 (5.1%)	0 (0%)	16 (11.7%)	12 (8.8%)	18 (6.5%)	42 (15.1%)	8 (8.1%)	20 (20.2%)
	Sparse	0 (0%)	0 (0%)	9 (6.6%)	6 (4.4%)	30 (10.8%)	25 (9%)	10 (10.1%)	18 (18.2%)
	Moderate	1 (2.6%)	1 (2.6%)	6 (4.4%)	4 (2.9%)	27 (9.7%)	13 (4.7%)	6 (6.1%)	9 (9.1%)
	Frequent	20 (51.3%)	15 (38.5%)	60 (43.8%)	24 (17.5%)	76 (27.2%)	48 (17.2%)	16 (16.2%)	12 (12.1%)
		p = 0.23		p = 0.12		p < 0.001		p = 0.03	
Hippocampal Tangle Density	None	1 (2.6%)	0 (0%)	3 (2.2%)	5 (3.6%)	1 (0.4%)	6 (2.2%)	0 (0%)	3 (3%)
	Sparse	1 (2.6%)	0 (0%)	3 (2.2%)	8 (5.8%)	7 (2.5%)	11 (4%)	2 (2%)	9 (9%)
	Moderate	0 (0%)	0 (0%)	6 (4.3%)	5 (3.6%)	13 (4.7%)	21 (7.6%)	0 (0%)	7 (7%)
	Frequent	21 (53.9%)	16 (41%)	80 (57.6%)	29 (20.9%)	130 (46.8%)	89 (32%)	28 (38%)	41 (41%)
		p = 0.23		p < 0.001		p = 0.001		p = 0.002	
Entorhinal Tangle Density	None	0 (0%)	0 (0%)	1 (0.7%)	1 (0.7%)	0 (0%)	1 (0.4%)	0 (0%)	0 (0%)
	Sparse	1 (2.6%)	0 (0%)	1 (0.7%)	4 (2.8%)	1 (0.4%)	6 (2.2%)	0 (0%)	1 (1%)
	Moderate	1 (2.6%)	0 (0%)	5 (3.6%)	3 (2.2%)	3 (1.1%)	16 (5.8%)	1 (1%)	10 (10%)
	Frequent	21 (53.9%)	16 (41%)	85 (61.2%)	39 (28.1%)	147 (53.1%)	103 (45.5%)	38 (38%)	50 (50%)
		p = 0.23		p = 0.08		p < 0.001		p = 0.02	

n (% = percent).

When analyzed by individual ApoE genotype, cases containing at least 1 ϵ4 allele (the 2/4, 3/4, and 4/4 groups) had greater frequencies of “frequent” ratings for both plaque and tangle scores when compared to the 2/3 and 3/3 groups. Within the ApoE ϵ4 carriers, the proportion of “frequent” ratings tended to increase (2/4 < 3/4 < 4/4). For all ApoE genotypes, the distribution of plaque pathology scores was skewed toward the “moderate” and “frequent” ratings for the frontal, temporal, and parietal areas (Table 5). For the entorhinal and hippocampal areas, there was a more even distribution of plaque scores among the ApoE genotypes. All groupwise comparisons showed statistically significant differences (p< 0.001) with all ϵ4 carriers having greater pathology than non-carriers and for increased pathology within the ϵ4 carrier group (2/4 < 3/4 < 4/4).

**Table 5:** Frequency of plaque pathology scores for all ApoE genotypes

		**2/3**	**3/3**	**2/4**	**3/4**	**4/4**
Frontal Plaque Density	None	0 (0%)	1 (0.4%)	0 (0%)	0 (0%)	0 (0%)
	Sparse	1 (4.5%)	4 (1.7%)	0 (0%)	0 (0%)	1 (0%)
	Moderate	5 (22.7%)	28 (21.4%)	1 (7.7%)	11 (4.7%)	2 (3.3%)
	Frequent	16 (72.7%)	198 (85.7%)	12 (92.3%)	224 (95.3%)	57 (96.7%)
Temporal Plaque Density	None	0 (0%)	0 (0%)	0 (0%)	0 (0%)	0 (0%)
	Sparse	1 (4.5%)	6 (2.6%)	0 (0%)	0 (0%)	0 (0%)
	Moderate	5 (22.7%)	30 (13.1%)	1 (7.7%)	15 (6.4%)	1 (1.7%)
	Frequent	16 (72.7%)	193 (84.3%)	12 (92.3%)	221 (93.6%)	59 (98.3%)
Parietal Plaque Density	None	0 (0%)	2 (0.9%)	0 (0%)	0 (0%)	0 (0%)
	Sparse	0 (0%)	3 (1.3%)	0 (0%)	0 (0%)	0 (0%)
	Moderate	5 (22.7%)	22 (9.6%)	0 (0%)	6 (3%)	3 (5%)
	Frequent	17 (77.3%)	201 (88.2%)	13 (100%)	229 (97%)	56 (95%)
Hippocampal Plaque Density	None	6 (27.3%)	20 (8.8%)	1 (7.7%)	4 (1.7%)	0 (0%)
	Sparse	6 (27.3%)	54 (23.7%)	3 (23.1%)	28 (11.9%)	5 (8.3%)
	Moderate	7 (31.8%)	81 (35.5%)	3 (23.1%)	108 (46%)	22 (36.7%)
	Frequent	3 (13.6%)	73 (32%)	6 (46.1%)	95 (40.4%)	33 (55%)
Entorhinal Plaque Density	None	1 (4.5%)	8 (3.5%)	0 (0%)	3 (1.3%)	1 (1.7%)
	Sparse	5 (22.7%)	14 (6.1%)	2 (15.4%)	3 (1.3%)	0 (0%)
	Moderate	4 (18.2%)	75 (32.8%)	2 (15.4%)	59 (25%)	10 (16.7%)
	Frequent	12 (54.5%)	132 (57.6%)	9 (69.2%)	171 (72.4%)	49 (81.7%)

n (% = percent).

For tangle scores, the overall distribution of score frequencies was somewhat more even than plaque scores (Table 6); however the ApoE ϵ4 carries tended to show greater tangle pathology than non-carriers across all cortical areas. For both plaque and tangle scores, all groupwise comparisons showed statistically significant differences (p< 0.005) with the exception of parietal tangle count where ApoE 3/3 and ApoE 3/4 p = 0.56 and hippocampal tangle count where ApoE 3/3 and ApoE 3/4 p = 0.98. ϵ4 carriers showed greater tangle pathology than non-carriers. Increased tangle pathology within the ϵ4 carrier group was also noted (2/4 < 3/4 < 4/4).

**Table 6:** Frequency of tangle pathology scores for all ApoE genotypes

		**2/3**	**3/3**	**2/4**	**3/4**	**4/4**
Frontal Tangle Density	None	9 (4.1%)	57 (24.7%)	2 (15.4%)	35 (15%)	4 (6.7%)
	Sparse	3 (13.6%)	62 (26.8%)	4 (26.7%)	39 (16.7%)	12 (20%)
	Moderate	3 (13.6%)	22 (9.5%)	2 (15.4%)	31 (5.6%)	8 (13.3%)
	Frequent	7 (31.8%)	90 (39%)	5 (38.5%)	129 (55.1%)	36 (60%)
Temporal Tangle Density	None	4 (18.2%)	25 (10.9%)	2 (15.4%)	17 (7.2%)	0 (0%)
	Sparse	6 (27.3%)	46 (20.1%)	2 (15.4%)	22 (9.4%)	7 (11.7%)
	Moderate	2 (9%)	34 (14.8%)	1 (7.7%)	19 (8.1%)	7 (11.7%)
	Frequent	10 (45.5%)	124 (54.1%)	8 (61.5%)	177 (75.3%)	46 (76.7%)
Parietal Tangle Density	None	10 (45.5%)	64 (28.2%)	3 (23.1)	36 (15.4%)	5 (8.5%)
	Sparse	2 (9%)	47 (20.7%)	3 (23.1)	36 (15.4%)	10 (5.9%)
	Moderate	2 (9%)	25 (11%)	1 (7.7%)	31 (13.2%)	8 (13.6%)
	Frequent	8 (36.4%)	91 (40.1%)	6 (46.1%)	131 (56%)	36 (61%)
Hippocampal Tangle Density	None	3 (13.6%)	11 (4.8%)	1 (8.3%)	4 (1.7%)	0 (0%)
	Sparse	1 (4%)	27 (11.8%)	0 (0%)	9 (3.8%)	4 (6.7%)
	Moderate	3 (13.6%)	30 (13.2%)	1 (8.3%)	17 (7.2%)	2 (3%)
	Frequent	15 (36.4%)	160 (70.2%)	10 (83.3%)	205 (87.2%)	54 (90%)
Entorhinal Tangle Density	None	1 (13.6%)	11 (4.8%)	1 (7.7%)	0 (0%)	0 (0%)
	Sparse	1 (4.5%)	27 (11.8%)	0 (0%)	3 (1.3%)	0 (0%)
	Moderate	1 (13.6%)	30 (13.2%)	1 (7.7%)	7 (3.0%)	2 (3.3%)
	Frequent	19 (0%)	160 (70.2%)	11 (84.6%)	223 (95.7%)	58 (96.7%)

n (% = percent).

## Discussion

In this study we found within a series of prospectively characterized and clinicopathologically confirmed AD cases, ApoE ϵ4 carriers had a significantly higher percentage of “frequent” scores for plaques and tangles when using the CERAD scale when compared to ApoE ϵ4 non-carriers in several regions of the brain. These results imply that carrying the ϵ4 allele increases the density of pathologies in several brain regions. Since AD is characterized by senile plaques and tangles, our study supports previous evidence demonstrating that the presence of the ApoE ϵ4 allele is a strong contributor to increased AD pathology. However, our study also demonstrates a much more detailed continuum of AD pathology as it relates to ApoE genotype. To our knowledge, the relationship between ApoE genotype and AD pathology has not been reported in this manner before. Previous studies have reported AD pathology and ApoE ϵ4 associations in terms of odds ratios [5,19]; however reporting this association in terms of CERAD score frequency may provide a more detailed picture of the differences in AD pathology severity between ApoE ϵ4 carriers and non-carriers. Additionally, we report this association as it relates to the distribution of CERAD pathology scores among different brain regions. In both ApoE ϵ4 carriers and non-carriers, we found that “sparse” and “moderate” plaque density scores were more prevalent in the hippocampus entorhinal regions when compared to the frontal, temporal, and parietal, regions in which “frequent” scores were more prevalent. Tangle density scores in the frontal, temporal, and parietal regions were more evenly distributed than plaque density scores within the ApoE ϵ4 carrier and non-carrier groups; however ApoE ϵ4 carriers had a higher prevalence of “frequent” scores for all brain regions when compared to non-carriers, except in the hippocampal and entorhinal areas among individuals age 90 and older where non-carriers had greater tangle pathology than carriers.

When analyzed by individual ApoE genotype, “none”, “sparse” and “moderate” plaque density scores were more prevalent among 3/3 carriers with 3/4 and 4/4 carriers having a higher prevalence of “frequent” scores among the different regions. A similar pattern was also noted for tangle density scores. There was a trend among all ApoE genotype groups for tangle pathology to be less frequent than plaque pathology in the frontal, temporal, and parietal regions. These results suggest that ApoE ϵ4 carries and non-carriers have independent neurodegenerative pathways in which plaque and tangle frequency vary among different cortical regions. Given that ApoE ϵ4 carrier status has been shown to result in differing clinical phenotypes of AD, [19-21] our results support these findings in demonstrating pathophysiologic differences between ApoE ϵ4 carriers and non-carriers.

Nagy et al. [1] found that, among all ApoE genotypes, 4/4 carriers displayed the greatest amount of AD pathology which is consistent with our results. In the current study, plaque and tangle pathologies increased in the genotype order 2/3<3/3<2/4<3/4<4/4 which has also been previously reported [5,6]. Ohm et al. [2] showed that the mean stages for beta amyloid deposition and Braak stages for NFTs were higher in those who carried the ApoE ϵ4 allele in comparison to non-carriers [2,5]. ApoE ϵ4 carriers not only had higher Braak NFT stages, but also an accelerated development of neurofibrillary changes [2,5]. Additionally, those who were homozygous for the ϵ4 allele had higher mean Braak stages than ϵ4 heterozygotes.

Beffert et al. [11] found that subjects with AD had decreased ApoE levels in both the hippocampus and frontal cortex. However, beta amyloid levels were significantly higher in AD cases compared controls. Those with the ApoE ϵ4 allele were found to have higher levels of Aβ (1–40 and 1–42) and lower levels of ApoE compared to non-carriers of the allele. From these results, Beffert et al. [11] suggest that lower ApoE levels are associated with increased Aβ levels.

Richey et al. [22] found that ApoE bound avidly to senile plaques and NFTs in AD brains, suggesting a direct interaction between ApoE and the aggregation of Aβ and tau. It has been suggested that increased ApoE levels may be the result of a neuroprotective mechanism that is triggered in response to the formation of NFTs [23]. If ApoE does play a protective role, then its common isoforms and their effects could vary in efficiency, with ApoE ϵ4 being the least efficient and ϵ2 the most. Beffert et al. [11] found decreased levels of ApoE in the hippocampus and the frontal cortex of AD brains which suggests that lower ApoE levels may make the brain more susceptible to the aggregation of AD pathology.

However, a study by Wisniewski et al. [24] showed that Aβ increases area ssociated with ApoE ϵ3 and ϵ4 in comparison to Aβ alone,with the ϵ4 isoform having the highest rate of increase in Aβ production. This implicates ApoE’s role as an accelerator for Aβ formation. It was also shown in vitro that the carboxyl-terminus of ApoE could, itself, form amyloid-like fibrils, which were congo-red positive and are present in senile plaques, further emphasizing its role as a pathological chaperone [25]. Holtzman et al. [26] highlight the importance of ApoE lipidation status as it relates to amyloid deposition stating that decreases in ApoE lipidation lead to increased amyloid deposition through increased fibrillization of Aβ. In particular, ApoE ϵ4 is associated with greater Aβ fibrillization. Jiang et al. [27] state that ApoE lipidation status is important in terms of determining whether Aβ peptides are cleared from the brain or whether they fibrillize and become amyloid deposits.

Guo et al. [28] investigated the role of Aβ and its role as a potential neuroinflammatory stimulator in AD. The study found that ApoE ϵ3 and ApoE ϵ4 suppressed Aβ-induced endogenous ApoE levels, with ApoE ϵ4 having a more effective inhibitory action. However, it was shown that in the absence of Aβ, both of these ApoE isoforms stimulated cytokine interleukin-1β (IL-1β), a pro-inflammatory agent. Specifically, ApoE ϵ4 was associated with significantly more production of IL-1β than ApoE ϵ3. Guo et al. [28] concluded that overproduction of ApoE may trigger this particular pro-inflammatory response. It is also suggested that ApoE ϵ4 could be a less effective anti-inflammatory isoform compared to ϵ2 and ϵ3, explaining its association with higher risk for AD [29]. Others have suggested that ApoE ϵ4 is associated with increased Aβ deposition and compromised neural repair mechanisms which, in conjunction, are associated with increased risk and observed pathology in AD [30].

One positive aspect of this study is the large sample size which allowed for differences to be seen between the different ApoE genotypes. Although the CERAD scoring scheme is commonly used to quantify AD pathology, it uses a semi-quantitative scoring system and does not provide an exact measurement of pathological densities. One weakness of our study was the low number of ApoE 2/4 carriers relative to the other genotypes so the comparisons of pathology score frequencies to the other genotypes may be somewhat biased. However, since the estimated prevalence of the ApoE 2/4 isoform is relatively low (2%) [4] and since the ϵ2 allele confers the lowest risk of developing AD [4], it would appear to be difficult to obtain a large number of these cases. The low prevalence of ϵ2 carriers in the general population may also be the reason that no ApoE 2/2 carriers were present in the sample as previous studies have estimated ϵ2 prevalence at approximately 7 to 8 percent [14,31]. Also, the prevalence of ApoE ϵ4 carriers in this study was substantially higher than what would be expected in the general population [14]. A meta analysis conducted by Ward et al. [32] found that the pooled prevalence rate of ApoE ϵ4 carriers in AD studies was 48.7% (95% CI: 46.5% - 51.0%). This suggests that AD studies are likely to have higher proportions of ApoE ϵ4 carriers relative to the general population given the role of ApoE ϵ4 as a risk factor for AD. From these results, it is not surprising that our sample also had a high proportion of ApoE ϵ4 carriers.

Another point of consideration is whether or not these results are dependent upon the proportion of different clinicopathologic subtypes of AD. Murray et al. [19] report that up to 25% of pathologically confirmed AD cases may be those with atypical pathologic presentations (limbic predominant [LP], hippocampal sparing [HpSp]). This study also found that the LP and HpSp subtypes were associated with ApoE ϵ4 non-carrier status, however this association was not statistically significant.

Also, the geographical area from which tissue samples were collected is relatively homogenous with respect to ethnicity and socioeconomic status so it cannot be stated that these results would apply to populations that are more ethnically and intellectually diverse.

## Conclusion

The results of this study demonstrate that the density and cortical distribution of AD neuropathology differs between ApoE ϵ4 carriers and non-carriers. It is possible that these neuropathologic differences may play a role in determining clinical phenotype of AD. These results also suggest that different disease pathways are associated with ApoEϵ4 carrier status resulting in different distributions of AD neuropathology.

## Competing interests

The authors have no competing interests to disclose.

## Authors’ contributions

MNS participated in the study design, performed clinical assessments, made clinical diagnoses, and drafted the manuscript. MM participated in the study design, drafted the manuscript, and carried out the statistical analyses. BND drafted the manuscript and acquired tissue samples. KL participated in the study design and drafted the manuscript. LIS drafted the manuscript and acquired tissue samples. GS drafted the manuscript and acquired tissue samples. DGW performed ApoE genotyping and drafted the manuscript. KD performed clinical assessments and drafted the manuscript. SAJ performed clinical assessments, made clinical diagnoses, drafted the manuscript. TGB drafted the manuscript and acquired tissue samples. All authors read and approved the final manuscript.

## Pre-publication history

The pre-publication history for this paper can be accessed here:

http://www.biomedcentral.com/1471-2377/13/44/prepub
